# Pneumococcal pericarditis in a patient with newly diagnosed diabetes mellitus: a case report

**DOI:** 10.1186/s13256-022-03548-8

**Published:** 2022-09-29

**Authors:** Suzan Dijkstra, Jaco H. Houtgraaf, Sanjay U. C. Sankatsing

**Affiliations:** 1 Department of Internal Medicine, Diakonessenhuis, Bosboomstraat 1, 3582 KE Utrecht, The Netherlands; 2 Department of Cardiology, Diakonessenhuis, Bosboomstraat 1, 3582 KE Utrecht, The Netherlands

**Keywords:** Bacterial pericarditis, *Streptococcus pneumoniae*, Cardiac tamponade, Diabetes mellitus, Acute illness, Case report

## Abstract

**Background:**

After the introduction of antibiotics, pneumococcal pericarditis has become a rare finding. However, this severe condition with high mortality and complication rates requires rapid recognition and intervention. Herein, we describe a patient that presents with this rare disease resulting in an unusual, fatal outcome.

**Case presentation:**

A previously healthy, 68-year-old, Caucasian male presented with progressive fatigue, dyspnea, and appetite loss since 12 days. He was diagnosed with diabetes mellitus 5 days before presentation but had not started treatment. After echocardiography revealed pericardial effusion, pericardiocentesis was performed with immediate drainage of a large volume of purulent fluid suggestive of bacterial pericarditis. On the basis of cultures showing *Streptococcus pneumoniae* as the causative organism, a regimen of intravenous penicillin was initiated. Additionally, antidiabetic drugs were started as his diabetes also predisposed him to invasive infectious disease. No other primary source of the infection, such as pneumonia, was found. Though the patient was found to be severely ill on admission, his clinical condition improved. A total of 1235 mL of pericardial fluid was drained, and adequate drainage was confirmed by daily, bedside echocardiography. However, 6 days post-admission, the patient suddenly developed intrapericardial bleeding with blood clot formation on the right chamber with subsequent cardiac tamponade. With the blood clot precluding adequate drainage through the catheter, the patient suffered cardiac arrest and died before surgical intervention could be attempted.

**Conclusions:**

Pneumococcal pericarditis is a very rare but life-threatening disease that necessitates immediate intervention with antibiotics and drainage of the pericardial effusion. Thus, although symptoms may be variable and aspecific, early recognition of this condition is critical. The present case illustrates the presentation, diagnosis, and clinical course of a patient presenting with pneumococcal pericarditis in current clinical practice. Through this report, we aimed to increase awareness among clinicians both of the existence of this phenomenon and of its uncertain clinical course. As is highlighted by the case, patients with pneumococcal pericarditis are at high risk for complications and should be closely monitored.

**Supplementary Information:**

The online version contains supplementary material available at https://doi.org/10.1186/s13256-022-03548-8.

## Background

Since the advent of antibiotics in the early 1940s, bacterial pericarditis has become a rare clinical finding. Though *Streptococcus pneumoniae* used to be the most prevalent causative organism isolated, its occurrence has diminished after the introduction of penicillin and the pneumococcal conjugate vaccine [1, 2]. This pathogen most commonly causes infections of the respiratory tract or head and neck area, leading to pneumonia, otitis, or sinusitis. However, it can spread to sites that are usually considered sterile through contiguous extension from adjacent, infected tissues (such as the lung and pleura) or via hematogenous dissemination in bacteremia [3]. Pneumococcal pericarditis carries high mortality rates due to the primary—often severe—infection, the often immunocompromised host, the potential development of cardiac tamponade, and the frequently late recognition only after the development of hemodynamic instability [4]. Herein we describe a patient with newly diagnosed diabetes mellitus in whom purulent pericarditis caused by pneumococci led to the rapid evolution of cardiac tamponade and ultimately death despite adequate antibiotic therapy.

## Case presentation

**Fig. 1:**
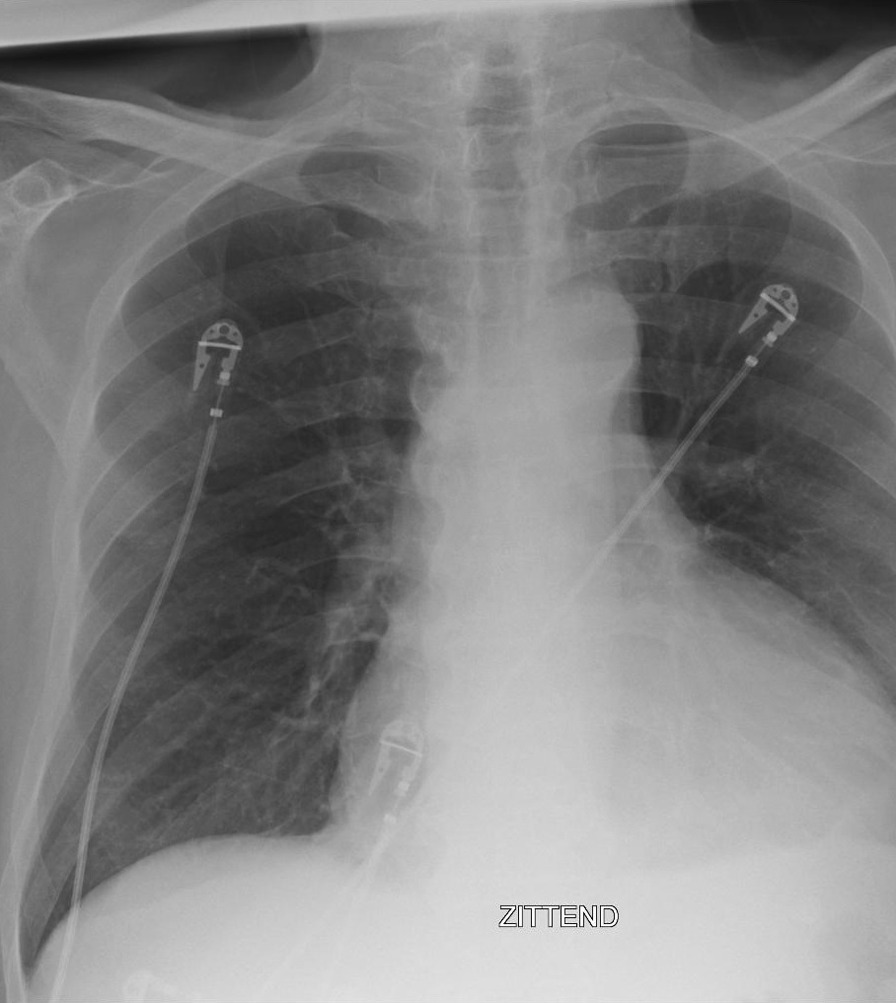
Chest X-ray showing an enlarged cardiac silhouette and left lower-lobe atelectasis with mild volume loss. The Dutch text in the X-ray image means “sitting”

**Fig. 2:**
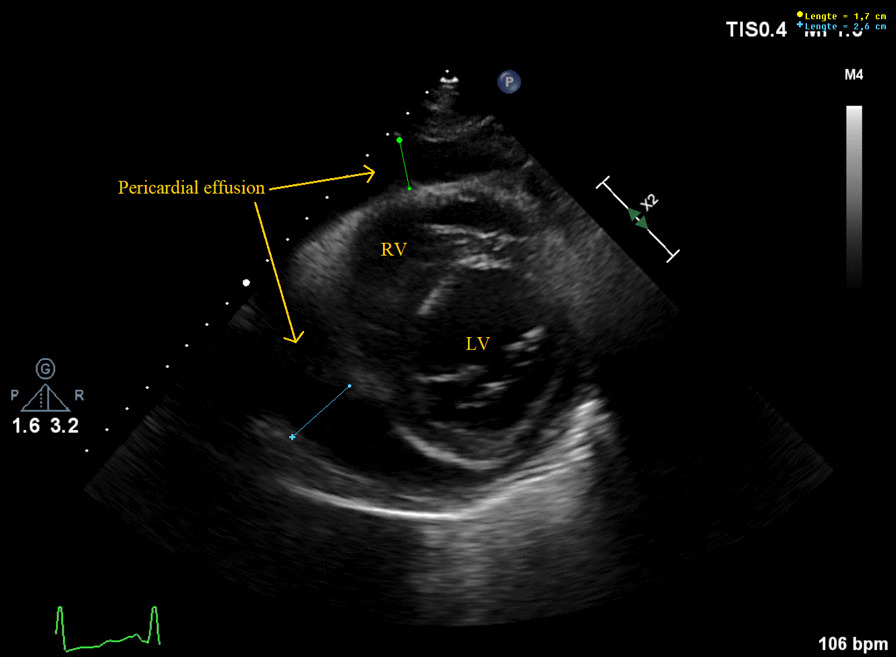
Echocardiogram upon admission (parasternal short-axis view) showing circumferential pericardial effusion. *LV* left ventricle, *RV* right ventricle

A 68-year-old Caucasian male was referred to the emergency department with progressive complaints of fatigue, dyspnea, and poor appetite since 12 days. He had also fallen down and needed help getting back up. The patient was a retiree who had previously worked in law enforcement. He had no significant past medical history and did not take regular medications. He had a long-term history of smoking (80 pack-years) and consumed three alcoholic drinks daily. His general practitioner had noted a new diabetes mellitus with a glucose of 28 mmol/L when the patient presented in his practice 5 days earlier, but had not started treatment. The patient did not report coughing, chills, or chest pain. On physical examination, he appeared dyspneic and had an altered mental status but could still speak complete sentences. He had a blood pressure of 141/105 mmHg, pulse rate of 115 beats per minute, respiratory rate of 34 breaths per minute, oxygen saturation of 97% without supplemental oxygen, and temperature of 36.2 °C. He had cold extremities with a capillary refill < 3 seconds. No other signs such as peripheral edema, heart murmurs, pericardial friction rub, or pulmonary crackles were present. Excepting the altered mental status, the global neurological examination was normal. Laboratory studies showed a C-reactive protein (CRP) level of 341 mg/L, neutrophilic leukocytosis with leukocyte count of 12.6 × 10^9^/L, glycated hemoglobin (HbA1c) of 96 mmol/mol, mildly elevated hepatic transaminases, and low serum myocardial markers. Arterial blood gas analysis revealed partially compensated respiratory alkalosis (pH 7.49, pCO_2_ 3.5 kPa, pO_2_ 12.6 kPa, HCO_3_ 19.4 mmol/L, base excess −2.4 mmol/L, sO_2_ 97%) (see Additional file 1 for all laboratory values at first presentation). Chest X-ray revealed an enlarged cardiac silhouette and left lower-lobe atelectasis but no evident pleural effusion or infiltration (Fig. 1). Electrocardiogram showed sinus tachycardia with a QS pattern in V1–4, suggestive of an old anterior wall myocardial infarction. Point-of-care echocardiography was performed and visualized large, circumferential pericardial effusion (Fig. 2). Also, wall motion abnormalities were seen that supported the theory that the patient had experienced an out-of-hospital anterior myocardial infarction in the weeks to months before the current presentation. The day after admission, ultrasound-guided pericardiocentesis was performed with immediate drainage of 600 mL of purulent fluid. Analysis of the fluid indicated a leukocyte count of 267 × 10^9^/L, total protein of 51 g/L, and lactate dehydrogenase pericardial fluid-to-serum ratio of 8.7. As Gram stains of blood revealed Gram-positive diplococci, antibiotic therapy consisting of continuous, intravenous infusion with 6 million international units benzylpenicillin daily was initiated. At admission, blood culture specimens for aerobic and anaerobic bacteria had been collected and directly transported to a BacT/Alert Virtuo system (BioMérieux). Later on, these blood cultures as well as pericardial fluid cultures grew *Streptococcus pneumoniae* (serotype unknown). Antimicrobial susceptibility tests showed that the isolated strain was susceptible to a variety of antibiotics including penicillin, with a minimum inhibitory concentration of 0.016 mg/L. Initially, insulin and metformin were started for the newly diagnosed diabetes mellitus. Acetylsalicylic acid and low-molecular-weight heparin where started to prevent occlusive vascular events. Moreover, during the admission, several other medications were started for cardiovascular protection, to treat electrolyte imbalances, and to supplement vitamins because of the previous alcohol use (see Additional file 2 for all medication administered during admission).

In the days following pericardiocentesis, a further 635 mL of purulent pericardial fluid was drained through the pericardial catheter and the patient’s clinical condition improved markedly. Daily, bedside transthoracic echocardiography confirmed adequate drainage of the pericardial effusion. The patient did not develop any symptoms that pointed us in the direction of a primary focus (for instance, no coughing, urinary complaints, or back pains). Repeated chest X-rays did not show signs of pneumonia or empyema. Human immunodeficiency virus (HIV) antigen/antibody assay was negative, as were tests for other microorganisms (ELISpot for detection of *M. tuberculosis*; hepatitis C virus serology; and feces assays for *C. difficile*, *Campylobacter*, *Salmonella*, *Shigella*, *Yersinia*, and norovirus). The CRP level dropped to 102 mg/L. However, 6 days post-admission, the patient’s condition suddenly deteriorated with a blood pressure of 100/66 mmHg, sinus tachycardia of 131 beats per minute, and respiratory rate of 33 breaths per minute. A small amount of sanguineous fluid drained through the pericardial catheter. Echocardiography revealed reaccumulation of pericardial fluid including blood clot formation at the level of the right chamber in the pericardial cavity. Moreover, the wide inferior vena cava, right ventricular compression, and increased respiratory inflow variation (> 25%) over the mitral valve were indicative of cardiac tamponade. Attempts to clear the drain blockage by flushing with saline and adjusting the catheter’s location in the pericardial cavity did not lead to evacuation of any pericardial fluid. Thus, as removal of the obstructing blood clot through the pericardial catheter proved impossible, the patient was transferred to a tertiary hospital to undergo emergent cardiothoracic surgery. Immediately before transfer, the patient went into cardiac arrest and he was ultimately transported with ongoing cardiopulmonary resuscitation. Unfortunately, surgical removal of the clot proved unsuccessful, and the patient died only hours after onset of his sudden hemodynamic deterioration. In consultation with the family, no autopsy was performed.

## Discussion

Herein, we report a case of pneumococcal pericarditis in an adult patient with rapid and severe clinical deterioration resulting in death. In addition to the rarity of the condition itself, three specific aspects of this case are noteworthy: (1) the untreated diabetes mellitus as a predisposing risk factor, (2) the absence of another primary infection focus, and (3) the sudden intrapericardial bleeding.

Nontuberculous bacterial pericarditis is reported to constitute < 1% of all cases of acute pericarditis [5]. Though *S. pneumoniae* previously accounted for half of these cases [1], pneumococcal pericarditis has become a rare phenomenon since the introduction of antimicrobials. Previously published reports on this condition are mainly case reports and small series. Most reports regarding data on incidence, diagnosis, and mortality predominantly rely on information from studies conducted prior to 1995. However, as healthcare is constantly evolving with improved treatment effectiveness, monitoring, and implementation of point-of-care echocardiography in the emergency department, these data may no longer be representative of the current situation. In a relatively recent prospective cohort study (patients enrolled between 1998 and 2001), only three out of 844 hospitalized patients with *S. pneumoniae* bacteremia developed pericarditis [6]. All three had comorbidities or risk factors for serious infectious disease (that is, HIV, corticosteroid use, or intravenous drug use). In our patient, his untreated diabetes mellitus and smoking predisposed him to invasive pneumococcal disease [7]. He had no other underlying conditions, such as malignancy, HIV infection, chronic autoimmune diseases, use of immunosuppressants, preexisting pericardial effusion, or recent thoracic surgery [8]. However, as the pneumococcal vaccine was introduced in the Dutch national immunization program for children as recently as 2006, it is also unlikely that he received this kind of prophylaxis for pneumococcal disease. As pneumococcal vaccines have been demonstrated to lower the incidence of vaccine-type invasive pneumococcal disease and pneumococcal pneumonia [9], it is intuitively attractive to assume that the addition of these vaccines to national immunization plans will lower the incidence of pneumococcal pericarditis as well.

*Streptococcus pneumoniae* infections are known to spread both contiguously from adjacent tissues or via bacteremia. Most commonly, pneumococcal pericarditis is a serious complication of a pleuropulmonary infection. Primary pneumococcal pericarditis is very unusual. Nonetheless, our case is one of the few that have been reported in which no (other) primary source of infection was identified [10]. Though no ear examination was performed to evaluate the presence of a potential otitis, the patient did not complain of ear pain. Repeated chest X-rays did not show pulmonary involvement. However, Laaban and colleagues previously described a case in which pneumonia was seen on daily chest X-rays only after 14 days [11]. Similarly, in a recent case reported by Lopez Luis and colleagues, pneumonia was revealed only during autopsy [12]. As the follow-up in our patient was relatively short, a beginning pneumonia that was still too subtle to be radiologically apparent could be an underlying cause for the following pericarditis.

Pneumococcal pericarditis still carries a high mortality. Where invariably fatal if left untreated, mortality rates are estimated to be 15–40% [2, 13, 14] even with aggressive therapy with drainage and high-dose intravenous antibiotics. The prognosis is poor not only because it usually develops in the context of an already severe infection, but also because the clinical diagnosis is challenging to make and, thus, often delayed. Signs and symptoms can be noncharacteristic and may be attributed to the underlying infection, even when hemodynamic compromise develops [4]. In this case, the patient had already had physical complaints for 12 days before first presentation in the hospital. The definitive diagnosis of pneumococcal pericarditis was made with pericardial aspirate cultures. Alternatively, when patients have already been administered antibiotics and cultures remain negative, a pneumococcal urinary antigen assay or 16S ribosomal DNA sequencing of pericardial fluid can still indicate *S. pneumoniae* as the responsible pathogen [15]. With regard to the treatment, though pericardiocentesis may not immediately influence the choice of antibiotic, early source control through effective drainage of the pericardial effusion is promptly warranted [13]. Furthermore, the development of a large pericardial effusion can lead to sudden cardiac tamponade. Even after adequate drainage, reaccumulation of fluid can occur rapidly, as purulent effusions can be viscous and loculated [13], and ongoing inflammation may promote constrictive pericarditis [16]. Thus, both surgical pericardiotomy/pericardiectomy and intrapericardial fibrinolysis should be considered to facilitate complete drainage of the effusion [13, 16, 17]. In this case, although appropriate antibiotic therapy and pericardiocentesis were commenced 1 day after presentation, intrapericardial bleeding resulted in obstruction of the pericardial catheter by a blood clot. To date, few cases of purulent pericarditis and concomitant bleeding have been reported, both in the absence [18] or presence [19] of a mycotic aneurysm. In our patient, the following sudden recurrence of cardiac tamponade led to hemodynamic deterioration and cardiac arrest before surgical drainage could be attempted.

## Conclusions

Purulent pericarditis, an extremely rare manifestation of invasive pneumococcal disease, still carries high mortality rates. As timely drainage and initiation of appropriate antimicrobials are of paramount importance for improving outcomes, the possibility of pneumococcal pericarditis should be considered in patients presenting with critical infectious illness, especially when pleuropulmonary involvement or an enlarged cardiac silhouette are observed. However, the purpose of this case report is also to show that, even with prompt therapy, pneumococcal pericarditis remains a serious disease with a high likelihood of clinical deterioration or complications even after initial improvement. Thus, all patients with pneumococcal pericarditis warrant very close clinical and echocardiographic monitoring. The publication of further reports will provide more awareness on the clinical significance of this condition as well as up-to-date experiences with current treatment options.

## Supplementary Information

**Additional file 1.** Laboratory values at first presentation.

**Additional file 2.** Medication administered from admission until the sudden clinical deterioration.

## Data Availability

Data sharing is not applicable to this article as no datasets were generated or analyzed during the current study.

## Abbreviations

CRP: C-reactive protein
HbA1c: Glycated hemoglobin
HIV: Human immunodeficiency virus
