# Supplementary material for: Pneumococcal pericarditis in a patient with newly diagnosed diabetes mellitus: a case report
Source: J Med Case Rep. 2022 Sep 29;16:344. doi: 10.1186/s13256-022-03548-8 (PMC9520949; doi:10.1186/s13256-022-03548-8)
Supplement: Supplementary file 1 — Additional file 1. Laboratory values at first presentation. [file 13256_2022_3548_MOESM1_ESM.pdf]

Additional file 1. Laboratory values at first presentation

	Venous blood	Arterial blood	Urine
CRP (mg/L)	341	-	-
Hemoglobin (mmol/L)	8.4	-	-
Hematocrit (L/L)	0.42	-	-
MCV (fl)	102	-	-
Thrombocytes (x10^9/L)	240	-	-
Leukocytes (x10^9/L)<br>Microscopic differential:<br>- Basophils (x10^9/L)<br>- Eosinophils (x10^9/L)<br>- Neutrophils (x10^9/L)<br>- Lymphocytes (x10^9/L)<br>- Monocytes (x10^9/L)<br>- Immature neutrophils (%)<br>- Toxic granulation<br>- Hypersegmentation	12.6<br><br>0.0<br>0.0<br>10.9<br>1.1<br>0.6<br>1<br>1+<br>present	-	-
Creatinine (umol/L)	71	-	-
Urea (mmol/L)	11	-	-
Sodium (mmol/L)	129	-	-
Potassium (mmol/L)	6.2	-	-
Total bilirubin (umol/L)	10	-	-
Alkaline phosphatase (U/L)	145	-	-
GGT (U/L)	55	-	-
ALT (U/L)	148	-	-
AST (U/L)	245	-	-
Lipase (U/L)	89	-	-
LD (U/L)	524	-	-
CK (U/L)	39	-	-
Troponin-T (ug/L)	0.020	-	-
Glucose (mmol/L)	31.6	-	-
pH	7.29	7.49	-
pCO2 (kPa)	6.7	3.5	-
pO2 (kPa)	4.8	12.6	-
HCO3 (mmol/L)	23.4	19.4	-
BE (mmol/L)	-3.6	-2.4	-
sO2 (%)	55	97	-
Ketones	-	-	2+
Sediment analysis:<br>- Erythrocytes (/uL)<br>- Leukocytes (/uL)			 77<br>27

Abbreviations: ALT, alanine transaminase; AST, aspartate transaminase; BE, base excess; CK, creatine kinase; CRP, C-reactive protein; GGT, gamma-glutamyl transferase; HCO3, bicarbonate; LD, lactate dehydrogenase; MCV, mean corpuscular volume; pCO2, partial pressure of carbon dioxide; pH, potential of hydrogen; pO2, partial pressure of oxygen; sO2, oxygen saturation.
