# Supplementary material for: Pneumococcal pericarditis in a patient with newly diagnosed diabetes mellitus: a case report
Source: J Med Case Rep. 2022 Sep 29;16:344. doi: 10.1186/s13256-022-03548-8 (PMC9520949; doi:10.1186/s13256-022-03548-8)
Supplement: Supplementary file 2 — Additional file 2. Medication administered from admission until the sudden clinical deterioration. [file 13256_2022_3548_MOESM2_ESM.pdf]

Additional file 2. Medication administered from admission until the sudden clinical deterioration

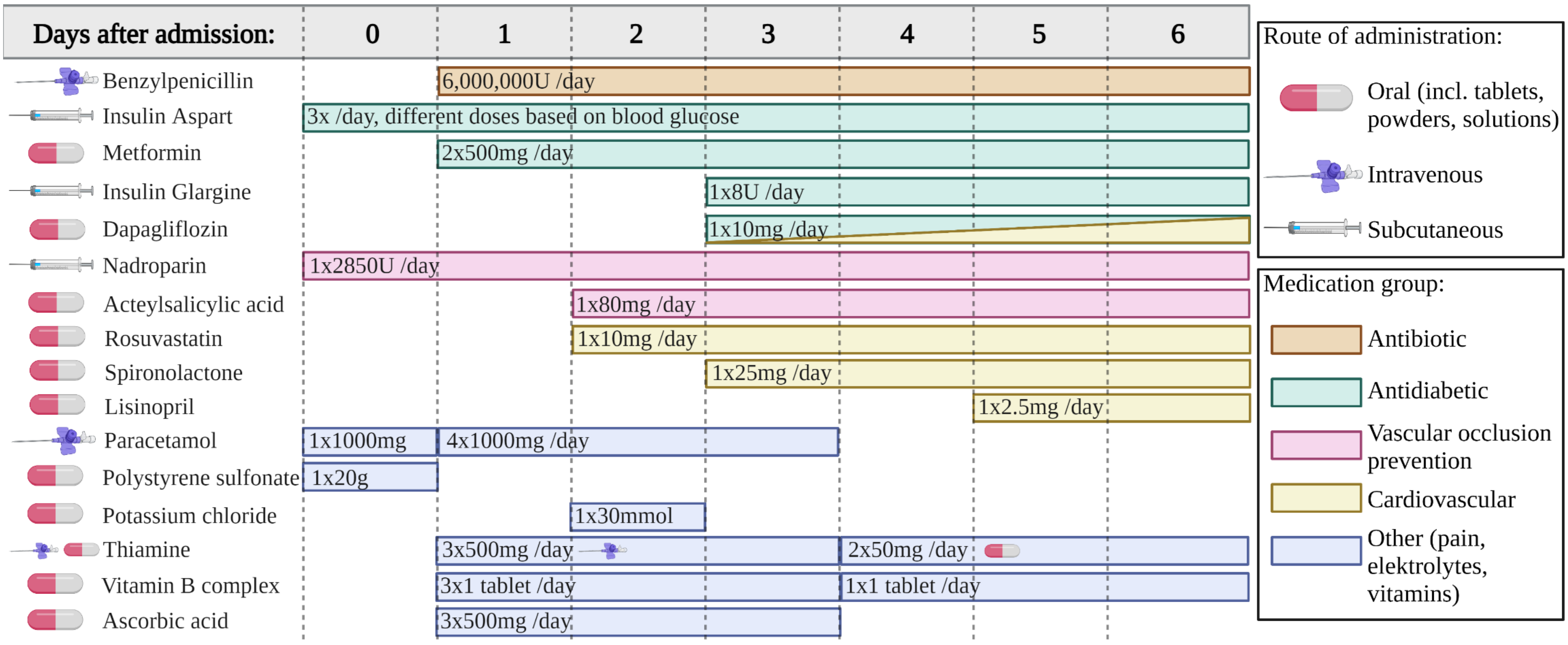

Abbreviations: g, gram; incl., including; mg, milligram; U, units.
Figure created with BioRender.com.
